# Effect of Remote Ischemic Preconditioning Evaluated by Nurses on Improvement of Arterial Stiffness, Endothelial Function, Diastolic Function, and Exercise Capacity in Patients with Heart Failure with Preserved Ejection Fraction (PIRIC-FEp Study): Protocol for Randomised Controlled Trial

**DOI:** 10.3390/biomedicines13081923

**Published:** 2025-08-07

**Authors:** Iris Otero Luis, Alicia Saz-Lara, Arturo Martinez-Rodrigo, María José Rodríguez-Sánchez, María José Díaz Valentín, María José Simón Saiz, Rosa María Fuentes Chacón, Iván Cavero Redondo

**Affiliations:** 1 CarVasCare Research Group, Faculty of Nursing, University of Castilla-La Mancha, 16071 Cuenca, Spain; iris.otero@uclm.es (I.O.L.); mariajose.rodriguez@uclm.es (M.J.R.-S.); MariaJose.Diaz@uclm.es (M.J.D.V.); ivan.cavero@uclm.es (I.C.R.); 2 COMETA Research Group, Informatics Systems Department, University of Castilla-La Mancha, 16071 Cuenca, Spain; arturo.martinez@uclm.es; 3 Hospital Virgen de la Luz, 16002 Cuenca, Spain; 4 Landete Health Center, 16330 Cuenca, Spain; 5 Department of Nursing, University of Castilla-La Mancha, 16071 Cuenca, Spain; mjose.simon@uclm.es (M.J.S.S.); rosa.fuentes@uclm.es (R.M.F.C.)

**Keywords:** heart failure with preserved ejection fraction, remote ischemic preconditioning, exercise capacity, cardiac function, endothelial function, arterial stiffness

## Abstract

**Background/Objectives**: Heart failure with preserved ejection fraction (HFpEF) has increased in prevalence as the population ages and associated comorbidities increase. Remote ischemic preconditioning (RIPC) has been shown to provide protection against ischemic injury to the heart and other organs. Therefore, the aim of this project will be to analyse the effectiveness of RIPC in terms of arterial stiffness, endothelial function, diastolic function, and exercise capacity in patients with HFpEF. **Methods**: The PIRIC-FEp study will be a parallel, randomised controlled trial with two groups conducted at the Faculty of Nursing in Cuenca, University of Castilla-La Mancha. Individuals who are diagnosed with HFpEF and are older than 40 years, with a left ventricular ejection fraction ≥50% and a sedentary lifestyle, will be included. The exclusion criteria will include, among others, patients with noncardiac causes of heart failure symptoms, significant pulmonary disease, diabetes, peripheral vascular disease, or myocardial infarction within the previous three months. A sample size of 48 patients was estimated, with 24 for each group. Participants will be randomly allocated (1:1) to either the RIPC intervention group or the control group to evaluate the effects on arterial stiffness, endothelial function, diastolic function, and exercise capacity. Assessments will be conducted at baseline and after a three-month follow-up period. **Results**: The findings will be published in a peer-reviewed journal article. **Conclusions**: This study is important for daily clinical practice because it provides a new approach for the treatment of HFpEF patients via RIPC.

## 1. Introduction

Heart failure (HF) is a clinical syndrome with various underlying aetiologies. HF can be classified into several types on the basis of the ejection fraction (EF) range, including heart failure with reduced ejection fraction (HFrEF), characterised by an EF less than or equal to 40%, and heart failure with preserved ejection fraction (HFpEF), where the EF is greater than 50% [1]. In HFpEF, the heart is unable to supply the amount of oxygen needed by the tissues or does so only at the expense of an excessive increase in the left ventricular ejection fraction within the normal range [2,3].

HF has been described as a pandemic, affecting 64.3 million people worldwide according to the most recent data from 2017 [4]. It has an incidence of 840 cases per 100,000 people annually, a prevalence of 3.4%—a figure that is expected to rise—and an annual global mortality rate of 24% among adults [5]. In the last decade, approximately 50% of patients with HF have been diagnosed with HFpEF, which generally has a better prognosis than HFrEF [3,6]. The prevalence of HFpEF has increased in parallel with population ageing and the increase in metabolic disorders such as obesity, diabetes, and hypertension [6].

Remote ischaemic preconditioning (RIPC) is a noninvasive cardioprotective intervention involving brief cycles of limb ischaemia and reperfusion. RIPC has the potential to protect the heart and other organs from damage caused by lethal ischaemia and reperfusion in several clinical settings [7]. Evidence supports the potential of RIPC to further improve exercise capacity, cardiac function, endothelial function, and arterial stiffness [8].

Several systematic reviews and meta-analyses of randomised controlled trials (RCTs) have investigated the effects of RIPC on a range of surgical interventions, both cardiac and vascular [9,10,11,12,13,14,15,16,17,18,19,20,21,22,23,24,25,26,27], as well as other surgical procedures [28,29,30], and in patients with a history of stroke [31,32]. These reviews examined outcomes such as troponin levels [9,16,21,29,30], myocardial injury biomarkers [11,15,20], arrhythmias [15,24,26,30], myocardial infarctions [14,15,17,18,20,21,22,23,24,25,26,27,28,30], myocardial injury [23,28,30], cardiac events [14,18,29], and stroke [17,25,30,32]. Length of stay in the intensive care unit [12,17] and overall hospitalisation time [9,12,13,30] were also evaluated. However, none of these reviews assessed the effects of RIPC on exercise capacity, endothelial function, or arterial stiffness.

Despite the substantial number of clinical trials examining the effects of RIPC in various pathologies and interventions, none have been conducted in patients with HFpEF. This may be due to the complex pathophysiology of this condition, as well as the wide heterogeneity of symptoms and comorbidities [33]. Therefore, we will hypothesise that RIPC may represent an effective treatment to reduce the morbidity and mortality associated with HFpEF. Consequently, this randomised controlled trial (RCT) will be conducted with the following objectives: (1) to evaluate the efficacy of nurse-administered RIPC—a noninvasive cardioprotective intervention using cycles of limb ischemia and reperfusion—in improving arterial stiffness, endothelial function, diastolic function, and exercise capacity, and (2) to assess its impact on quality of life (QoL) and its cost-effectiveness compared with conventional treatment.

## 2. Materials and Methods

### 2.1. Design

The PIRIC-FEp study will be a 2-arm, parallel-group RCT evaluating an RIPC intervention in patients with HFpEF over a 3-month period. The design of this study allows for the evaluation of the effectiveness of the therapeutic intervention and minimises selection bias. By including two parallel groups, one intervention group and one control group, treatment crossover is avoided, and the internal validity of this study is reinforced, facilitating a direct and reliable comparison between the two groups. This RCT protocol has been registered on ClinicalTrials.gov (NCT07024810).

### 2.2. Setting

Patient recruitment for this RCT will be performed in the primary healthcare centres of Cuenca, as well as in the cardiology and internal medicine services of the Virgen de la Luz Hospital of Cuenca, belonging to the Castilla-La Mancha Health Service (SESCAM). Healthcare professionals from these centres and services will be informed of the trial aims to support the recruitment of eligible HFpEF patients. These patients are subsequently invited to participate in the RCT through information sessions, posters, media announcements, and referrals from healthcare professionals.

### 2.3. Participants

Patients diagnosed with HFpEF in accordance with the 2021 ESC guidelines [34] will be eligible for inclusion. The inclusion and exclusion criteria are presented in Table 1.

### 2.4. Randomisation

**Figure 1:**
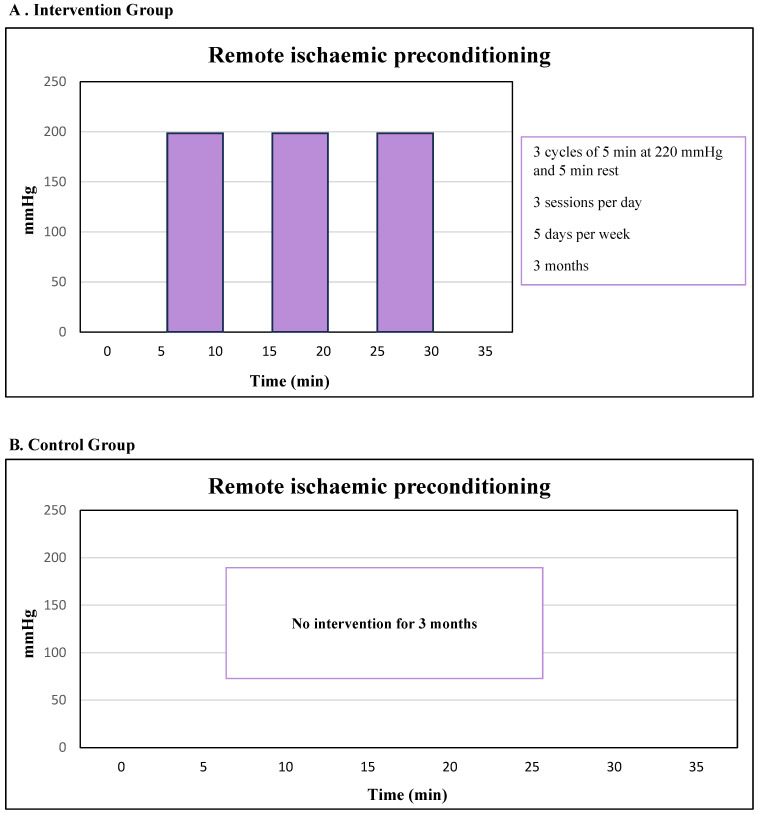
Remote ischaemic preconditioning (RIPC) and control group (CG) protocols.

Participants will be randomly assigned (1:1) via EPIDAT version 4.2 software (Figure 1) to one of two groups: Group 1, RIPC; Group 2, the control group (CG). Prior to randomisation, screening will be performed, and all participants will be required to sign the informed consent form.

### 2.5. Intervention

#### 2.5.1. Remote Ischaemic Preconditioning (RIPC)

Participants randomised to the RIPC intervention group will be provided with a portable blood pressure monitor (Welch Allyn DuraShock™ DS45, Skaneateles Falls, NY, USA) to perform the RIPC procedure themselves. The cuff will be positioned on the upper arm and inflated to 220 mmHg for 5 min, followed by 5 min of deflation. This sequence will be repeated three additional times. The procedure will be performed five days per week for a period of three months. The RIPC will be applied to the left arm (with the exception of any condition that prevents it, in which case it will be performed on the right arm). The participants will be supervised during their first RIPC session and every 15 days thereafter to ensure proper administration. Participants will be allowed to carry out the RIPC procedure at any time during the day and will document each session in a diary to track adherence. They will be advised to continue with their regular daily activities and avoid starting new exercise routines or making changes to their diet. In the event of any adverse effects, such as pain, tissue damage [37], haematomas [37,38], numbness or paraesthesia [39], acute deep vein thrombosis, or acute ischaemia of the upper limb [38], the guidelines outlined in Table S1 will be followed.

#### 2.5.2. Control Group

Participants randomly assigned to the RIPC intervention group will receive a hand-held blood pressure monitor.

Each participant in the CG will be instructed to continue with their usual routine and avoid engaging in any new physical activities or dietary changes.

#### 2.5.3. Duration of the Intervention

**Figure 2:**
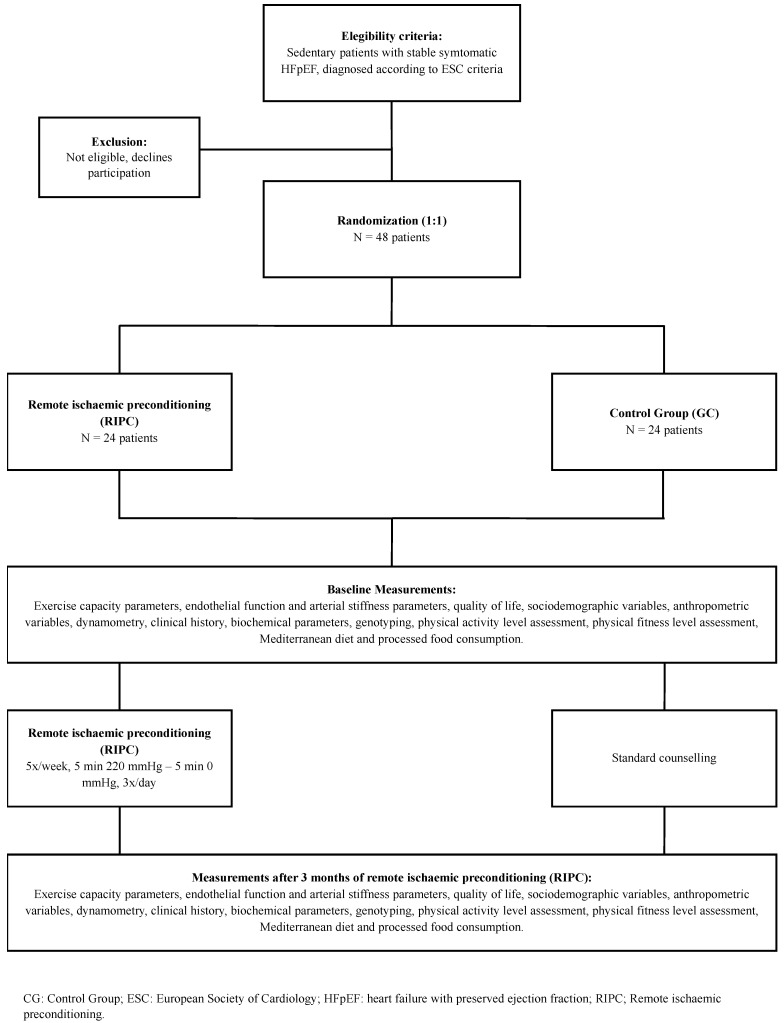
Flowchart for patient inclusion and follow-up: screening, inclusion and exclusion, randomisation, baseline testing, and postintervention testing.

The intervention period will span three months. Figure 2 presents the flowchart detailing patient inclusion and follow-up steps, including screening, eligibility criteria, randomisation, baseline assessments, and evaluations after the intervention.

### 2.6. Nonadherence Criteria and Extension of the Intervention

Patients will be considered adherent to the RIPC protocol if they complete a minimum of 70% of the planned sessions within the specified period. Compliance with the intervention will be monitored and documented periodically throughout this study via weekly phone calls and biweekly visits. Should a participant need to pause RIPC due to unforeseen reasons during the three-month intervention, the duration may be extended by up to four additional weeks.

### 2.7. Outcomes

All outcomes will be collected and assessed by registered nurses who are members of the research team; however, they will not be blinded, as they will also be responsible for monitoring compliance and instructing patients on the application of the RIPC.

#### 2.7.1. Primary Outcomes

Exercise capacity:

- Six-Minute Walking Test (6MWT): This test will be conducted in a 30 m long corridor. The participants will walk back and forth along this section, which will be marked with cone-shaped markers placed 29 m apart, leaving 0.5 m at each end to facilitate turns. The test will be supervised by the examiner.

Endothelial function:

- Carotid Intima-Media Thickness (CIMT): CIMT will be assessed via ultrasound using the Sonosite SII device, Bothell, WA, USA. CIMT is an independent predictor of future clinical events such as myocardial infarction and sudden cardiac death [40] and allows for the assessment of structural changes over time, facilitating the monitoring of atherosclerosis [41].

Arterial stiffness:

- Pulse wave velocity (PWV), the radial augmentation index (rAIx), and the central augmentation index (cAIx): These measurements will be obtained via the SphygmoCor System (AtCor Medical Pty Ltd., West Ryde, NSW, Australia) while the patient is lying in a supine position. Pulse waves will be recorded from the carotid and femoral arteries. The rAIx is calculated as follows: (Second systolic peak [SBP2] − Diastolic blood pressure [DBP]) divided by (First systolic peak − DBP), multiplied by 100 (%). The cAIx is calculated as follows: central pulse pressure augmentation times 100 divided by pulse pressure. All indices will be derived directly from the respective devices.

#### 2.7.2. Covariates

Sociodemographic variables and medical history were collected via self-reports.

Sociodemographic Variables

- Age.
- Sex.
- Socioeconomic status: Participants reported their socioeconomic and employment status. These factors will be used to calculate an index based on the Spanish Society of Epidemiology scale.

Medical history

- Comorbidities: Chronic conditions such as hypertension, diabetes, COPD, smoking, and alcohol use will be recorded.
- Pharmacological treatment: Pharmacological therapies followed by each patient will be collected through questionnaires.

Anthropometric Variables

- Weight: Weight will be recorded as the mean of two measurements using a Seca^®^ 861 scale, with participants barefoot and in light clothing.
- Height: The average of two measurements will be taken using a wall-mounted stadiometer (Seca^®^ 222, Hamburg, Germany), with participants standing barefoot, upright, and with their sagittal midline aligned to the stadiometer.
- Body mass index (BMI): BMI will be calculated via the following formula: weight (kg) divided by height squared (m^2^).
- Waist circumference: The average of three measurements will be recorded via a flexible tape measure at the midpoint between the lowest rib and the iliac crest, taken at the end of a normal exhalation.
- Body fat percentage: The mean of two assessments will be obtained via the Tanita^®^ BC-418 MA eight-electrode bioelectrical impedance (Tanita Corp., Tokyo, Japan).
- Blood pressure: We will record the mean of two readings taken 5 min apart using an Omron^®^ HEM-907 monitor (Omron Healthcare Milton Keynes, UK Ltd.), after a 5 min rest, with the patient seated in a quiet environment and the right arm semiflexed at heart level. Cuffs of three sizes will be used depending on the arm circumference.

Muscle strength:

- Handgrip strength will be measured using a TKK 5401 grip-D dynamometer (Takei^®^, Tokyo, Japan). Two measurements will be taken on each arm, and the average will be calculated. The hand size will be taken into consideration to adjust the dynamometer grip accordingly.

Step Cadence, Heart Rate, Sleep Quality and Duration, and METs (Metabolic Equivalents):

- Variables such as step cadence (steps/min), METs (activity-related energy expenditure), and heart rate (beats/min) will be continuously recorded at 1 min intervals via the Fitbit Inspire 3 activity tracker, which will be worn for 9 days.
- Aggregated metrics of sleep quality and duration will be recorded daily on the basis of Fitbit data, providing a comprehensive assessment of participants’ sleep patterns.

Biochemical parameters

Blood samples will be collected from an antecubital vein between 8:15 and 9:00 a.m. after a minimum 12 h fast. Three aliquots will be frozen: one for the purposes of the current study and two for potential future analyses (with the participants’ informed consent).

- The following biomarkers will be evaluated: glucose, total cholesterol, triglycerides, HDL and LDL cholesterol, apolipoproteins A1 and B, insulin, and high-sensitivity C-reactive protein (hsCRP). These analyses will be conducted via the Cobas 8000 system (Roche Diagnostics^®^), while insulin levels will be measured on the Architect platform (Abbott^®^).
- HbA1c Glycated haemoglobin will be analysed via high-performance liquid chromatography (HPLC) via the ADAMS A1c HA-8180 V analyser (A. Menarini Diagnostics^®^), which is certified by both the NGSP and the IFCC.

Genotyping:

- Genotyping will be performed using a saliva sample collected in a sterile tube mixed with a preservative reagent. A sufficient volume (1–3 mL) is required to ensure adequate DNA content for analysis.

Physical Activity:

- International Physical Activity Questionnaire (IPAQ): The IPAQ assesses physical activity by classifying it into three intensity levels and calculates weekly energy expenditure in MET minutes.

Physical Fitness:

- International Fitness Scale (IFIS): A validated self-report assessment questionnaire was used to assess perceived physical fitness.

Diet:

- Mediterranean Diet Adherence Screener (MEDAS-14): A 14-item validated questionnaire will be used to evaluate adherence to the Mediterranean dietary pattern.
- Processed Food Consumption Questionnaire (SQ-HPF): A validated questionnaire evaluating the frequency and quantity of intake in 14 food categories will be used.

Quality of Life:

- Short Form Health Survey (SF-12);
- Minnesota Living with Heart Failure Questionnaire (MLWHFQ).

### 2.8. Ethical Considerations

The study protocol received approval from the Clinical Research Ethics Committee of the Hospital Virgen de la Luz in Cuenca, the site where the research will be carried out (REG: 2025/PI0625).

In compliance with Spanish Law 3/2018 of 5 December on the Protection of Personal Data and Guarantee of Digital Rights (LOPD-GDD), the rights and freedoms of study subjects will be respected, including the confidentiality of their data.

Participants will be informed at every stage of this study regarding its objectives and methodology and will be asked to provide written informed consent.

### 2.9. Sample Size Calculation

The sample size was calculated using Epidat 4.2 software, which is based on the data reported by Maxwell et al. [42]. To achieve 90% statistical power and a significance level of 0.05, a sample size of 20 patients per group is needed to detect a statistically significant difference in flow-mediated dilatation. Assuming a 20% dropout rate, the final sample size is estimated at 48 participants (24 in each group).

### 2.10. Statistical Analysis

Statistical analysis will be performed in three phases: verification of the success of randomisation in creating comparable groups of patients with HFpEF; exploration of outliers and extreme values; and assessment of the normality of the primary variables.

Analysis of covariance (ANCOVA) models will be used, with each outcome variable as the dependent variable and intervention as the fixed effect (1 = RIPC; 0 = CG), adjusted for baseline values, medication, age, sex, and socioeconomic status. The results will be presented as absolute changes from baseline to follow-up, along with 95% confidence intervals (CIs). Pairwise comparisons will be adjusted via the Bonferroni post hoc correction.

As a sensitivity analysis, a propensity score matching (PSM) approach will be applied to account for imbalances in baseline covariates between groups. This causal inference model estimates what would have occurred if all the subjects in both groups had shared the same baseline characteristics. Each participant will be matched with other comparable characteristics via a 0.40 calliper through the psmatch2 command.

All analyses will follow the intention-to-treat principle, with participants analysed in the groups to which they were originally assigned, regardless of adherence, in line with the CONSORT guidelines.

A cost-effectiveness analysis of RIPC from a societal perspective will also be conducted. Costs over the 3-month period will be compared with the costs of usual care for patients with HFpEF. Effectiveness will be calculated as the difference in arterial stiffness between the RIPC and control groups.

Statistical significance will be set at *p* < 0.05. All analyses will be conducted via STATA version 16.

### 2.11. Mitigation Strategies

- Heterogeneity among participants.
  ○ To reduce heterogeneity among participants, the established inclusion and exclusion criteria will be rigorously applied, thereby ensuring a more homogeneous sample.

- Dropouts and missing data
  ○ Qualified nursing staff from the research team will be responsible for training participants in the correct application of the therapy, as well as monitoring follow-up diaries and providing reminders via weekly phone calls and biweekly visits to ensure adequate compliance and minimise losses during follow-up. In addition, analyses will be conducted according to the intention-to-treat principle, meaning that all participants will be included in the primary analysis, regardless of whether or not they complete the intervention. With respect to the treatment of missing data, appropriate statistical methods, such as imputation techniques, can be applied to reduce the bias associated with missing information.

- Small sample size.
  ○ Although the sample size is small, it has been calculated on the basis of previous estimates and a dropout rate of 20%, which has been incorporated into the calculation to ensure the minimal statistical power required to detect relevant effects.

- Exercise tolerance may be influenced by participants’ daily activities.
  ○ Participants will be instructed to maintain their usual lifestyle during their involvement in this study. To monitor this, measurements will be taken via activity trackers, the 6MWT, and self-reported physical activity questionnaires.

- Lack of blinding.
  ○ The absence of blinding in both participants and research staff could influence the subjectivity of some assessments. To mitigate this limitation, objective measures, such as anthropometric parameters, blood pressure, biochemical markers, muscle strength tests, and exercise capacity assessments, among others, will be included. Similarly, to reduce bias resulting from the lack of blinding in the research team, standardised protocols for data collection will be applied.

- Potential bias.
  ○ To mitigate potential bias, confounding variables such as medication, age, sex, and socioeconomic status will be considered in the analyses.

## 3. Results

The results will be published as a peer-reviewed article.

## 4. Discussion

The main aim of this study was to evaluate the effectiveness of RIPC in patients with HFpEF by assessing its impact on arterial stiffness, endothelial function, diastolic function, and exercise capacity. The secondary objectives are as follows: (1) To compare the effectiveness of RIPC on QoL in patients with HFpEF and (2) to analyse the cost-effectiveness of RIPC versus conventional treatment in patients with HFpEF.

### 4.1. RIPC, HFpEF, and Exercise Capacity

HFpEF is characterised by cardiovascular abnormalities such as myocardial stiffness and fibrosis, which may contribute to reduced exercise tolerance and symptoms such as oedema or chronotropic incompetence [43]. RIPC has been shown to be effective in improving exercise capacity in healthy individuals through both local (i.e., limb) and remote effects (via the cardiovascular or nervous system) [8]; however, it has not been explored in individuals with pathologies, such as HFpEF.

### 4.2. RIPC, HFpEF, and Cardiac Function

HFpEF is associated with abnormal cardiac function, including increased left ventricular stiffness, leading to impaired relaxation during diastole and resulting in elevated pressure and/or impaired filling. The effect of RIPC on cardiac function has been investigated in patients with coronary artery disease [44], in whom it was found to have a neutral effect. However, the influence of RIPC on cardiac function has rarely been explored and has not been studied in patients with HFpEF. Given the remote cardiovascular and neurological effects of RIPC, beneficial results from this intervention could be expected.

### 4.3. RIPC, HFpEF, and Endothelial Function

Endothelial dysfunction is considered an early marker in HFpEF, as it occurs early in the disease and manifests as a loss of cardiac microvascular density, which may be influenced by comorbidities commonly associated with HFpEF [45]. Previous studies have shown that RIPC can improve endothelial function via local and remote ischaemia-reperfusion mechanisms [46], and these effects may improve exercise capacity by preserving the supply of oxygen and energy substrates to skeletal muscles.

### 4.4. RIPC, HFpEF, and Arterial Stiffness

Arterial stiffness, particularly of the aorta, increases the load on the left ventricle, adversely affecting its structure and function. These changes contribute to the development of diastolic dysfunction, which is a key factor in HFpEF. Moreover, increased carotid–femoral pulse wave velocity has been associated with a greater risk of heart failure [47]. Given the contribution of arterial stiffness to HFpEF, interventions that target this parameter may prove beneficial in its management. To date, RIPC has shown inconclusive results in patients with peripheral arterial disease [48] and angina pectoris [42]; however, its effects have not yet been demonstrated in individuals with HFpEF.

### 4.5. Physiological Mechanisms of RIPC

RIPC contributes to the optimisation of mitochondrial function by increasing ATP synthesis, improving mitochondrial respiratory efficiency, reducing the opening of the mitochondrial permeability transition pore (mPTP), and decreasing the production of reactive oxygen species (ROS) [49]. Furthermore, it has been associated with improved vascular endothelial function and an increased release of nitric oxide, facilitating a reduction in both cardiac preload and afterload [50]. Additionally, evidence suggests that RIPC-induced mitochondrial improvement in T lymphocytes may protect the myocardium by exerting anti-inflammatory effects and mitigating ischaemia-reperfusion injury-related damage [51].

### 4.6. Potential Limitations

Some potential limitations of this study must be considered: (1) this study will be conducted with individuals from a single province (Cuenca), which limits the generalisability of the findings to the broader population; (2) although the proposed sample size is not very large, it may not be fully achieved, which would reduce the statistical power of this study; (3) the intervention will be carried out by the participants themselves following prior instructions provided by a healthcare professional. This could lead to errors in implementation and difficulties in monitoring adherence, potentially affecting the effectiveness of the RIPC. (4) The intervention will last three months, so even if effects are observed at the end of this period, they could be temporary. Therefore, longer-term studies will be necessary to assess the long-term effect of the RIPC; and (5) the absence of blinding in participants can affect subjective assessments, such as self-report questionnaires, and favour the emergence of biases associated with the placebo effect. For its part, the lack of blinding of the research team, in addition to affecting subjective assessments, could influence the way in which data are collected, recorded, or interpreted, thereby jeopardising the objectivity of this study.

## 5. Conclusions

This study has clinical relevance, as HFpEF has a high prevalence, which is expected to continue to increase in the coming years. This research will provide healthcare professionals with a novel approach to the treatment of HFpEF via RIPC, with the aim of improving functional capacity, cardiac function, endothelial function, and arterial stiffness.

## Figures and Tables

**Table 1:** Inclusion and exclusion criteria.

Inclusion Criteria	Exclusion Criteria
**1. Diagnosis of HFpEF (according to 2021 ESC criteria):** **Signs and symptoms of HF** **Left ventricular ejection fraction (LVEF) ≥ 50%** **Objective evidence of structural and/or functional cardiac abnormalities consistent with left ventricular diastolic dysfunction and/or elevated left ventricular filling pressures, including elevated natriuretic peptides** **2. Sedentary people (men and woman) (structured exercise <2 × 30 min/week)** **3. Age ≥ 40 years** **4. Written informed consent** **5. Clinically stable for at least 6 weeks** **6. On optimal medical therapy for ≥6 weeks**	1. Noncardiac causes of HF symptoms: - Significant valvular or coronary artery disease - Uncontrolled hypertension or arrhythmias - Primary cardiomyopathies 2. Significant pulmonary disease (FEV1 < 50% predicted, GOLD stage III–IV) 3. Inability to exercise or conditions that may interfere with the exercise intervention 4. History of myocardial infarction in the past three months 5. Patients with diabetes and/or peripheral vascular disease * 6. Signs of ischaemia during maximal cardiopulmonary exercise testing 7. Comorbidities that may affect 1-year prognosis 8. Enrolment in a different clinical study

ESC: European Society of Cardiology. HF: Heart failure. HFpEF: Heart failure with preserved ejection fraction. FEV1: Forced expiratory volume in 1 s. GOLD: Global Initiative for Chronic Obstructive Lung Disease. * Exclusion criterion 5 specifies that patients with diabetes and/or peripheral vascular disease were excluded. In the case of type 2 diabetes, this decision was based on evidence indicating that RIPC does not consistently confer cardioprotective effects in this population. This limitation is attributed to mitochondrial dysfunction caused by elevated levels of glycosylated proteins in diabetic patients, which interferes with the protective mechanisms typically activated by RIPC [35]. With regard to peripheral vascular disease, patients were excluded because the application of RIPC is contraindicated due to the risk of local ischaemia or tissue damage in the affected limbs [36].

## Supplementary Materials

The following supporting information can be downloaded at https://www.mdpi.com/article/10.3390/biomedicines13081923/s1. Table S1. Guidelines for managing adverse effects of Remote Ischaemic Preconditioning in Patients Enrolled in the PIRIC-FEp Study.

## Abbreviations

The following abbreviations are used in this manuscript:

CG	Control group
EF	Ejection fraction
HF	Hear failure
HFpEF	Heart failure with preserved ejection fraction
HFrEF	Heart failure with reduced ejection fraction
QoL	Quality of life
RCT	Randomised controlled trial
RIPC	Remote ischaemic preconditioning

## References

[1] Shahim B., Kapelios C.J., Savarese G., Lund L.H. (2023). Global Public Health Burden of Heart Failure: An Updated Review. Card. Fail. Rev..

[2] Leggio M., Fusco A., Armeni M., D’Emidio S., Severi P., Calvaruso S., Limongelli G., Sgorbini L., Bendini M.G., Mazza A. (2018). Pulmonary hypertension and exercise training: A synopsis on the more recent evidence. Ann. Med..

[3] Cavero-Redondo I., Saz-Lara A., Martínez-García I., Bizzozero-Peroni B., Díaz-Goñi V., Díez-Fernández A., Moreno-Herráiz N., Pascual-Morena C. (2023). Comparative Effect of Two Types of Physical Exercise for the Improvement of Exercise Capacity, Diastolic Function, Endothelial Function and Arterial Stiffness in Participants with Heart Failure with Preserved Ejection Fraction (ExIC-FEp Study): Protocol for a Randomised Controlled Trial. J. Clin. Med..

[4] Savarese G., Becher P.M., Lund L.H., Seferovic P., Rosano G.M.C., Coats A.J.S. (2022). Global burden of heart failure: A comprehensive and updated review of epidemiology. Cardiovasc. Res..

[5] Cavero-Redondo I., Saz-Lara A., Bizzozero-Peroni B., Núñez-Martínez L., Díaz-Goñi V., Calero-Paniagua I., Matínez-García I., Pascual-Morena C. (2024). Accuracy of the 6-Minute Walk Test: ExIC-FEp Trial and a Meta-Analysis. Sports Med. Open.

[6] Omote K., Verbrugge F.H., Borlaug B.A. (2022). Heart failure with preserved ejection fraction: Mechanisms and treatment strategies. Annu. Rev. Med..

[7] Healy D.A., Clarke Moloney M., McHugh S.M., Grace P.A., Walsh S.R. (2014). Remote ischaemic preconditioning as a method for perioperative cardioprotection. Int. J. Surg..

[8] Sharma V., Marsh R., Cunniffe B., Cardinale M., Yellon D.M., Davidson S.M. (2015). From Protecting the Heart to Improving Athletic Performance. Cardiovasc. Drugs Ther..

[9] D’Ascenzo F., Cavallero E., Moretti C., Omedè P., Sciuto F., Rahman I.A., Bonser R.S., Yunseok J., Wagner R., Freiberger T. (2012). Remote ischaemic preconditioning in coronary artery bypass surgery: A meta-analysis. Heart.

[10] Marczak J., Nowicki R., Kulbacka J., Saczko J. (2012). Is remote ischaemic preconditioning of benefit. Interact. Cardiovasc. Thorac. Surg..

[11] Yetgin T., Manintveld O.C., Boersma E., Kappetein A.P., van Geuns R.J., Zijlstra F., Duncker D.J., van der Giessen W.J. (2012). Remote ischemic conditioning in PCI and CABG. Circ. J..

[12] Li L., Li G., Yu C., Li Y. (2013). The role of remote ischemic preconditioning on postoperative kidney injury. J. Cardiothorac. Surg..

[13] Zhou C., Liu Y., Yao Y., Zhou S., Fang N., Wang W., Li L. (2013). β-blockers and volatile anaesthetics may attenuate cardioprotection. J. Cardiothorac. Vasc. Anesth..

[14] D’Ascenzo F., Moretti C., Omedè P., Cerrato E., Cavallero E., Er F., Presutti D.G., Colombo F., Crimi G., Conrotto F. (2014). Remote ischaemic preconditioning reduces periprocedural MI. EuroIntervention.

[15] Healy D.A., Khan W.A., Wong C.S., Moloney M.C., Grace P., Coffey J., Dunne C., Walsh S., Sadat U., Remote Preconditioning Trialists’ Group (2014). Remote preconditioning and major complications. Int. J. Cardiol..

[16] Yang L., Wang G., Du Y., Ji B., Zheng Z. (2014). Remote ischemic preconditioning reduces troponin I. J. Cardiothorac. Vasc. Anesth..

[17] Benstoem C., Stoppe C., Liakopoulos O.J., Ney J., Hasenclever D., Meybohm P., Goetzenich A. (2017). Remote ischaemic preconditioning for CABG. Cochrane Database Syst. Rev..

[18] Wang S., Li H., He N., Sun Y., Guo S., Liao W., Liao Y., Chen Y., Bin J. (2017). Impact of RIPC in CV surgery. Int. J. Cardiol..

[19] Zhou C., Bulluck H., Fang N., Li L., Hausenloy D.J. (2017). Age and surgical complexity impact on renoprotection. Sci. Rep..

[20] Deferrari G., Bonanni A., Bruschi M., Alicino C., Signori A. (2018). Remote ischaemic preconditioning in cardiac surgery. Nephrol. Dial. Transplant..

[21] Xie J., Zhang X., Xu J., Zhang Z., Klingensmith N.J., Liu S., Pan C., Yang Y., Qiu H. (2018). RIPC in adult cardiac surgery. Anesth. Analg..

[22] De Freitas S., Hicks C.W., Mouton R., Garcia S., Healy D., Connolly C., Thomas K.N., Walsh S.R. (2019). Ischemic Preconditioning in AAA repair. J. Surg. Res..

[23] Stather P.W., Wych J., Boyle J.R. (2019). RIPC for vascular surgery: Systematic review. J. Vasc. Surg..

[24] Zhang M.H., Du X., Guo W., Liu X.P., Jia X., Wu Y. (2019). RIPC after AAA repair. Vasc. Endovasc. Surg..

[25] Chen E., Cai W., Hu D., Chen L. (2020). RIPC in STEMI during PCI. Rev. Cardiovasc. Med..

[26] Ouyang H., Zhou M., Xu J., Fang C., Zhong Z., Zhou Y., Xu J., Zhou W. (2020). RIPC in elective vascular surgery. Ann. Vasc. Surg..

[27] Long Y.Q., Feng X.M., Shan X.S., Chen Q.C., Xia Z., Ji F.H., Liu H., Peng K. (2022). RIPC reduces AKI after cardiac surgery. Anesth. Analg..

[28] Lamidi S., Baker D.M., Wilson M.J., Lee M.J. (2021). RIPC in noncardiac surgery. J. Surg. Res..

[29] Wahlstrøm K.L., Bjerrum E., Gögenur I., Burcharth J., Ekeloef S. (2021). RIPC and mortality after noncardiac surgery. BJS Open.

[30] Fresilli S., Labanca R., Turi S., Casuale V., Vietri S., Lombardi G., Covello R.D., Lee T.C., Landoni G., Greco M. (2025). RIPC and survival in noncardiac surgery. Br. J. Anaesth..

[31] Jiang B., Wang X., Ma J., Fayyaz A., Wang L., Qin P., Ding Y., Ji X., Li S. (2024). RIC after stroke. CNS Neurosci. Ther..

[32] Li Q., Guo J., Chen H.S., Blauenfeldt R.A., Hess D.C., Pico F., Khatri P., Campbell B.C., Feng X., Abdalkader M. (2024). RIC in ischemic stroke: Meta-analysis. Neurology.

[33] Hobbach A., Brix T., Weyer-Elberich V., Varghese J., Reinecke H., Linke W. (2025). Obesity and comorbidities in HFpEF: A retrospective cohort analysis in a university hospital setting. J. Clin. Med..

[34] McDonagh T.A., Metra M., Adamo M., Gardner R.S., Baumbach A., Böhm M., Burri H., Butler J., Čelutkienė J., Chioncel O. (2021). ESC Guidelines on heart failure. Eur. Heart J..

[35] Costa J.F., Fontes-Carvalho R., Leite-Moreira A.F. (2013). Myocardial remote ischemic preconditioning: From pathophysiology to clinical application. Rev. Port. Cardiol. (Engl. Ed.).

[36] Zhao W., Meng R., Ma C., Hou B., Jiao L., Zhu F., Wu W., Shi J., Duan Y., Zhang R. (2017). Safety and efficacy of remote ischemic preconditioning in patients with severe carotid artery stenosis before carotid artery stenting: A proof-of-concept, randomized controlled trial. Circulation.

[37] Beltman F.W., Heesen W.F., Smit A.J., May J.F., I Lie K., Jong B.M.-D. (1996). Acceptance and side effects of ambulatory blood pressure monitoring: Evaluation of a new technology. J. Hum. Hypertens..

[38] Healy D., Clarke-Moloney M., Gaughan B., O’Daly S., Hausenloy D., Sharif F., Newell J., O’donnell M., Grace P., Forbes J.F. (2015). Preconditioning Shields Against Vascular Events in Surgery (SAVES), a multicentre feasibility trial of preconditioning against adverse events in major vascular surgery: Study protocol for a randomised control trial. Trials.

[39] Casazza F., Bongarzoni A., Guenzati G., Tassinario G., Mafrici A. (2015). Fulminant pulmonary embolism successfully treated with thrombolysis. Analg. Resusc. Curr. Res..

[40] Chambless L.E., Folsom A.R., Clegg L.X., Sharrett A.R., Shahar E., Nieto F.J., Rosamond W.D., Evans G. (2000). Carotid wall thickness is predictive of incident clinical stroke: The Atherosclerosis Risk in Communities (ARIC) study. Am. J. Epidemiol..

[41] Groot E., Leuven S., Duivenvoorden R., Meuwese M., Akdim F., Bots M., Kastelein J. (2008). Measurement of carotid intima–media thickness to assess progression and regression of atherosclerosis. Nat. Clin. Pract. Cardiovasc. Med..

[42] Maxwell J.D., Carter H.H., Hellsten Y., Miller G.D., Sprung V.S., Cuthbertson D.J., Thijssen D.H.J., Jones H. (2019). Seven-day remote ischaemic preconditioning improves endothelial function in patients with type 2 diabetes mellitus: A randomised pilot study. Eur. J. Endocrinol..

[43] Zuo L., Chuang C.C., Hemmelgarn B.T., Best T.M. (2015). ROS and NO in HFpEF. J. Appl. Physiol..

[44] Nagueh S.F. (2020). LV diastolic function via echocardiography. JACC Cardiovasc. Imaging.

[45] Simmonds S.J., Cuijpers I., Heymans S., Jones E.A.V. (2020). HFpEF vs HFrEF pathology. Cells.

[46] Epps J., Dieberg G., Smart N.A. (2016). Repeat RIPC and cardiovascular function. Int. J. Cardiol. Heart Vasc..

[47] Mitchell G.F. (2021). Arterial stiffness in aging. Hypertension.

[48] Zagidullin N., Scherbakova E., Safina Y., Zulkarneev R., Zagidullin S. (2016). RIPC in angina pectoris. J. Clin. Med..

[49] Kleinbongard P., Gedik N., Kirca M., Stoian L., Frey U., Zandi A., Thielmann M., Jakob H., Peters J., Kamler M. (2018). Mitochondrial and contractile function of human right atrial tissue in response to remote ischemic conditioning. J. Am. Heart Assoc..

[50] Kepler T., Kuusik K., Lepner U., Starkopf J., Zilmer M., Eha J., Vähi M., Kals J. (2020). El preacondicionamiento isquémico remoto atenúa los biomarcadores cardíacos durante la cirugía vascular: Un ensayo clínico aleatorizado. Eur. J. Vasc. Endovasc. Surg..

[51] Zhao Z., Lv M., Li X., Li M. (2025). Impact of remote ischemic preconditioning on the T-lymphocyte mitochondrial damage index: A randomized clinical trial. Signa Vitae.

