# Supplementary material for: Effect of Remote Ischemic Preconditioning Evaluated by Nurses on Improvement of Arterial Stiffness, Endothelial Function, Diastolic Function, and Exercise Capacity in Patients with Heart Failure with Preserved Ejection Fraction (PIRIC-FEp Study): Protocol for Randomised Controlled Trial
Source: Biomedicines. 2025 Aug 7;13(8):1923. doi: 10.3390/biomedicines13081923 (PMC12383716; doi:10.3390/biomedicines13081923)
Supplement: Supplementary file 1 [file biomedicines-13-01923-s001.zip › biomedicines-3741670-supplementary.pdf]

Supplementary Material

Table S1. Guidelines for managing adverse effects of Remote Ischaemic Preconditioning in Patients Enrolled in the PIRIC-FEp Study.

If any of these effects occur, the telephone number listed on the informed consent form signed at the start of the study should be contacted.

Averse event	Symtomps	Recommendation
Pain (37)	Discomfort or pain during inflation	Interrupt the intervention.<br>Notify the study coordinators.<br>If pain is severe, consider analgesia as prescribed by a physician.
Haematomas (37,38)	Change in skin colour to purple/black	Discontinue the intervention on the affected limb.<br>Apply local cold therapy.<br>Notify the study coordinators.<br>Have coagulation assessed by a specialist.
Tissue damage (37)	Appearance of a wound in the cuff pressure area	Discontinue the intervention on the affected limb.<br>Perform wound dressing.<br>Notify the study coordinators.<br>If the wound worsens, consult a specialist.
Numbness or paraesthesia (39)	Persistent tingling in the limb for more than 10 minutes	Interrupt the intervention.<br>Notify the study coordinators.<br>Evaluate neuropathy by a specialist.
Acute deep vein thrombosis (38)	Persistent pain, swelling, warmth, and redness in the affected area	Interrupt the intervention.<br>Consult a physician for assessment.<br>Notify the study coordinators.
Ischaemia of the upper limb. (38)	Persistent pain, loss of sensation, weakness, pallor of the limb, and weak pulse	Interrupt the intervention.<br>Consult a physician for assessment.<br>Notify the study coordinators.
